# Diabetic macular edema with pachychoroid features

**DOI:** 10.1186/s12886-020-01663-y

**Published:** 2020-10-02

**Authors:** Kyungeun Kang, Hyungwoo Lee, Minsu Jang, Hyung Chan Kim, Hyewon Chung

**Affiliations:** Department of Ophthalmology, Konkuk University School of Medicine, Konkuk University Medical Center, 120-1 Neungdong-ro, Gwangjin-gu, Seoul, 05030 Republic of Korea

**Keywords:** Diabetic retinopathy, Diabetic macular edema, Pachychoroid, Central macula thickness, Subfoveal choroidal thickness

## Abstract

**Background:**

To investigate the clinical features of diabetic macular edema (DME) in eyes with pachychoroid phenotypes using multimodal retinal imaging.

**Methods:**

We retrospectively reviewed 210 eyes from 210 DME patients and analyzed the clinical and imaging parameters, including visual acuity, central macular thickness (CMT), subfoveal choroidal thickness (SFCT) and neural retina layer thickness (NRT). The DME eyes were divided into two groups: group 1 (80 eyes with submacular detachment [SMD]) and group 2 (130 eyes without SMD). The clinical and imaging parameters of 285 eyes from 285 diabetic patients without DME were collected as a control group.

**Results:**

DME eyes with pachychoroid phenotypes were more frequent in group 1 than in group 2 (53 eyes [66.25%] and 53 eyes [40.77%], respectively, *P* < 0.001)**.** Pachychoroid phenotypes were identified in 108 (37.90%) of the control eyes. CMT and NRT were greater in group 1 than in group 2. In group 1, 37 eyes had SMD combined with focal edema, and 43 eyes had SMD combined with diffuse-type edema. No significant difference in pachychoroid phenotypes was found between the focal and diffuse types (26 [70.27%] and 27 [62.79%], respectively, *P* = 0.481). In group 2, 70 eyes had focal-type edema, and 60 eyes had diffuse-type edema. No significant difference in the frequency of pachychoroid phenotypes was found (32 [45.71%] and 21 [35.00%], respectively, *P* = 0.215). Interestingly, among the 70 eyes with focal edema in group 2, 13 (40.6%) and 5 (13.2%) eyes with and without pachychoroid phenotypes showed no definite microaneurysms, respectively.

**Conclusion:**

SMD and focal edema without definite microaneurysms may be clinical manifestations of DME with pachychoroid phenotypes and possibly related to choroidal circulation disturbance in DME.

## Background

Diabetic macular edema (DME) is the most common cause of vision loss in diabetic retinopathy (DR), and its prevalence increases up to 28–29% in patients with a diabetes duration greater than 20 years [1]. Since Otani et al. [2] reported three patterns of DME, namely, diffuse swelling, cystoid macular edema (CME) and serous macular detachment (SMD), using optical coherence tomography (OCT), the clinical manifestations of each type of DME, including detailed morphologic classifications, have been described [3]. OCT can discriminate DME types, including those with SMD, which is difficult to diagnose by biomicroscopy or fluorescein angiography (FA) [4]. More recently, diffuse and focal edema have been described based on the different pathophysiologies of DME development: diffuse edema is thought to result from disrupted retinal vessel integrity induced by the generalized breakdown of the inner blood-retinal barrier (BRB) [5], whereas focal edema results from microaneurysms, with pathology observed primarily in the outer retina [6]. When diffuse leakage occurs in the inner retina, fluid collects mainly in the inner nuclear layer (INL), resulting in a diffuse edema pattern.

Among the various patterns of DME, the importance of SMD in diffuse or focal edema has not been adequately depicted. Although the prevalence of SMD is quite high, approximately ranging from 10 to 30% [2, 4, 7, 8], the pathogenesis and functional consequences of SMD remain controversial [4, 8, 9]. Inner and/or outer BRB compromise are the main causes for the development of macular edema in diabetic eyes; however, the genesis of SMD in these eyes seems involve a complex process. The inner plexiform layer (IPL) and outer plexiform layer (OPL) constitute 2 diffusion barriers of intraretinal fluid distribution [6]. The excessive intraretinal fluid from the disruption of the inner BRB has been hypothesized to reach the subretinal space because the external limiting membrane (ELM) is not impermeable to fluid and albumin [3, 10, 11]. If a large water load is not removed properly by the retinal pigment epithelium (RPE) (failure of the RPE pump), SMD could occur. One good example of SMD occurrence from inner BRB breakdown is in cases of retinal vein occlusion (RVO); the prevalence of SMD in RVO is much higher than that in DME. Excess fluid that reaches the subretinal space from rapidly increased intraretinal fluid in RVO might exceed the RPE active transport mechanism [2, 7]. However, this mechanism of SMD development from inner BRB disruption hardly explains the occurrence of SMD without substantial retinal fluid accumulation or when DME is focal (fluid in only the outer plexiform/outer nuclear layer [OPL/ONL]) and far from the macula [8], especially the nasal location. Thus, subretinal fluid accumulation may occur in DME with outer BRB breakdown, and choroidal circulation also leaks into the subretinal space.

There have been different reports regarding choroidal thickness (CT) during DR and DME. Some authors reported that the choroid thins during the progression of DR, reflecting decreased choroidal circulation and vascular insufficiency in DR and DME [12–16] whereas others reported no significant association between CT and DR or DME [17, 18]. In contrast, some authors found that CT increased with the severity of DR and in eyes with DME [19, 20]. There could be several explanations for these divergent reports, including not adjusting for age and refractive error and not using enhanced depth imaging OCT (EDI OCT), but studies of heterogeneous eyes with different prevailing pathophysiologies involved in the development of DME might be a major reason. Choroidal thickening is a manifestation observed in various disorders affecting the retina and choroid, including recently described pachychoroid spectrum diseases. Pachychoroid spectrum diseases, including pachychoroid pigment epitheliopathy (PPE), pachychoroid neovasculopathy (PNV), and central serous chorioretinopathy (CSC), have common characteristics: diffuse or focal areas of increased CT, dilated choroidal vessels (pachyvessels), and thinning or absence of inner choroid (the choriocapillaris [CC] and Sattler’s layer) overlying pachyvessels [21–25]. Although a number of studies have described various manifestations of these diseases, their pathophysiologies remain debatable. In particular, the influence of pachychoroid features on the manifestations of various forms of DME has not been studied.

Thus, this study was designed to determine the clinical manifestations of DME with or without pachychoroid features using multimodal retinal imaging. In this study, we investigated whether particular types of macular edema are more common in diabetic eyes with pachychoroid phenotypes.

## Methods

### Study population

A total of 2272 consecutive eyes with DR from 1136 diabetic patients who were followed up at Konkuk University Medical Center, Seoul, Korea, from November 2016 to October 2018 were included. For this retrospective study, we reviewed the medical records for 2272 eyes of 1136 patients. Only one eye was selected in each patient; if both eyes had DME, the first eye with this condition or the worse eye at presentation was selected. If patients had no macular edema in both eyes, the eye that had less media opacity and clearer images was selected. In 2272 eyes, 978 eyes were excluded because EDI OCT was not performed at the initial examination. A total of 444 eyes were excluded for the following reasons: vitreous hemorrhage, epiretinal membrane, vitreomacular traction and high myopia more than 6 diopters. A total of 298 eyes with a history of other eye diseases that may affect the outcome, such as RVO and age-related macular degeneration above the intermediate stage, were also excluded. In addition, we excluded 57 patients who had a history of uveitis, glaucoma, other previous intraocular surgery except for cataract and refractive surgery and past treatments such as laser photocoagulation or intravitreal injection. Finally, we included 285 eyes with no DME and 210 eyes with DME from among 495 eyes with DR.

A total of 495 eyes were divided into five groups by two retinal specialists. When the specialists disagreed while assigning the eyes to groups, they reviewed the patient charts together and reached a consensus. Eyes without DME were assigned to the control group. Group 1 was defined as DME with SMD. Group 1 was further divided into groups 1A and 1B according to the presence of focal or diffuse edema, respectively. As previously described [2, 6], focal edema was defined as retinal fluid collection only in the outer retina (the OPL to the ELM). In diffuse edema, retinal fluid is located in the inner retina (the internal limiting membrane [ILM] to the INL). Combined-type edema, where fluid is observed in the inner and outer retina, was also designated as diffuse edema. Thus, diffuse edema was defined as retinal fluid located in the inner retina with or without retinal fluid in the outer retina. Group 2 consisted of DME eyes without SMD. Group 2A was defined as focal-type edema, and group 2B was defined as diffuse-type edema. Finally, each group was further divided into two subgroups according to the presence or absence of pachychoroid features.

All patients underwent a comprehensive ophthalmological examination at the first visit, including measurement of the best-corrected visual acuity (BCVA) using a Snellen chart, intraocular pressure, slit-lamp biomicroscopy, fundus examination, and color fundus photography using Topcon TRC-50IX (Topcon Medical Systems Inc., Paramus, New Jersey, USA) or Optos P200Tx (Optos plc., Dunfermline, Scotland, UK). EDI OCT images were obtained using a Spectralis HRA + OCT® device (Spectralis; Heidelberg Engineering, Heidelberg, Germany). Some patients underwent other tests, including FA, ICGA, and OCT angiography (OCTA).

Pachychoroid was defined as a subfoveal choroidal thickness (SFCT) of 300 μm or more and at least one of the following characteristics based on previous studies [21–25]: the existence of enlarged choroidal vessels (pachyvessels) and/or attenuated inner choroid on EDI OCT or choroidal hyperpermeability on ICGA.

### Image analysis

Volume scans generated by SD OCT images at baseline were selected for analyses. These scans covered a 9 mm × 6 mm area and contained 25 scans spaced 238 μm apart. Our first goal of image analysis was to calculate the SFCT and inner and outer retina thicknesses. The choroid thickness was measured from the outer portion of the hyperreflective line corresponding to the RPE to the inner surface of the sclera on the EDI OCT image. Using the caliper tool available from Heidelberg, SFCT was measured perpendicularly 3000 μm wide in the subfoveal choroid centered on the fovea. The central macular thickness (CMT), inner retina layer (IPL/INL) and outer retina layer (OPL/ONL) were manually measured using manual segmentation. CMT was defined within a 1000 μm diameter, and inner and outer retina layer thicknesses were measured within the 3000 μm diameter cylinder at the macula. En face images of the CC were obtained by using OCTA with autosegmentation.

All of these processes were managed primarily by a trained ophthalmologist (K Kang) and further confirmed by a retinal specialist (H Chung).

### Statistical analyses

Statistical analyses were performed using IBM SPSS software ver. 18.0 for windows (IBM Corp, Chicago, IL, USA). Visual acuity obtained using a Snellen chart was switched to the logarithm of the minimal angle of resolution (logMAR) BCVA for statistical analyses. Pearson’s Chi-square test was performed to compare the ratio of sex and prevalence of pachychoroid, hypertension and dyslipidemia. Fisher’s exact test was performed to compare the prevalence of cardiovascular disease and kidney disease history. One-way ANOVA was performed to evaluate the difference in age. The Kruskal-Wallis test with the Mann-Whitney U test was used to compare the CMT, SFCT, BCVA (logMAR), IPL/INL thickness and OPL/ONL thickness. *P <* 0.05 was considered statistically significant.

## Results

This study included 495 eyes of 495 patients with DR (mean age 58.66 ± 12.69 years, 304 men and 191 women). The number of eyes with pachychoroid features in each group is described in Table 1. In the control group and groups 1A, 1B, 2A and 2B, 108 (37.9%), 26 (70.3%), 27 (62.8%), 32 (45.7%) and 21 (35.0%) eyes had pachychoroid phenotypes, respectively. The frequency of pachychoroid phenotypes was significantly higher in group 1 than in the control group (*P* < 0.001). The ratio of pachychoroid phenotypes was significantly higher in group 1 (66.3%) than in group 2 (40.8%, *P* < 0.001). The demographic characteristics of the patients from each group are shown in Table 1. There were no significant differences in age, sex, and the prevalence of hypertension, dyslipidemia, cardiovascular diseases and kidney diseases among the groups.

**Table 1:** Baseline characteristics of each group

	Control (***n*** = 285)	Group 1A (***n*** = 37)	Group 1B (***n*** = 43)	Group 2A (***n*** = 70)	Group 2B (***n*** = 60)	***p***-value	Total (***n*** = 495)
**Non-pachychoroid/Pachychoroid (number)**	177/108	11/26	16/27	38/32	39/21	< 0.001	281/214
**Sex (male/female)**	167/118	26/11	30/13	48/22	33/27	0.424	304/191
**Age (mean ± SD)**	59.41 ± 13.64	57.08 ± 11.35	55.74 ± 10.94	57.87 ± 11.30	59.05 ± 11.33	0.385	58.66 ± 12.69
**Prevalence of HTN (number, %)**	144 (50.5)	17 (45.9)	21 (48.8)	36 (51.4)	29 (48.3)	0.420	247 (49.9)
**Prevalence of dyslipidemia (number, %)**	56 (19.6)	5 (13.5)	8 (18.6)	14 (20.0)	6 (10.0)	0.429	89 (18.0)
**Prevalence of CVA Hx (number, %)**	42 (14.7)	6 (2.7)	2 (4.7)	6 (8.6)	5 (8.3)	0.185	61 (12.3)
**Prevalence of kidney disease Hx (number, %)**	19 (6.6)	4 (10.8)	6 (14.0)	8 (11.4)	4 (6.7)	0.376	41 (8.3)

Control, no diabetic macular edema (DME)

Group 1, serous macular detachment (SMD), further subdivided into group 1A with focal edema and group 1B with diffuse edema

Group 2A, focal edema type without SMD

Group 2B, diffuse edema type without SMD

*SD* standard deviation, *HTN* hypertension, *CVA Hx* cardiovascular disease history (including cerebral infarction, a history of cardiac arrest, angina, previous CABG, and previous PCI)

One-way ANOVA (age), Pearson’s Chi-square test (pachychoroid type, sex, hypertension, dyslipidemia) and Fisher’s exact test (CVA Hx, kidney disease Hx) were used

**Fig. 1:**
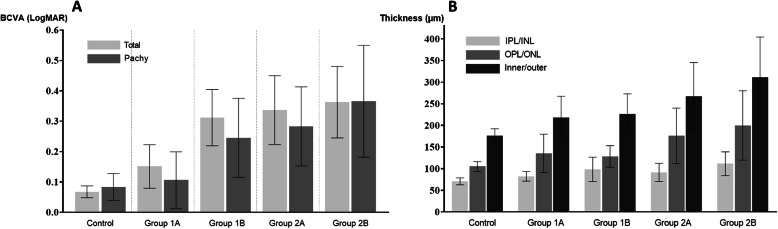
Comparison of baseline visual acuity and retinal layer thickness in pachychoroid-type patients in each group **a** Graphs showing the baseline BCVA in all patients and in pachychoroid-type patients. Among all groups (light gray bar graph), the mean logMAR BCVA at baseline was 0.16 ± 0.28. The mean BCVA for each group is as follows: 0.07 ± 0.17, 0.34 ± 0.34, 0.36 ± 0.38, 0.15 ± 0.30, and 0.31 ± 0.36 in the control group and groups 1A, 1B, 2A and 2B, respectively. In pachychoroid phenotypes (dark gray bar graph), the mean logMAR BCVA at baseline was 0.16 ± 0.31. The mean BCVA for each group is as follows: 0.08 ± 0.23, 0.28 ± 0.32, 0.37 ± 0.46, 0.11 ± 0.26 and 0.25 ± 0.29 in the control group and groups 1A, 1B, 2A and 2B, respectively. **b** Graphs showing the neural retinal thickness in pachychoroid-type patients. The mean thickness of the IPL/INL (light gray bar graph) was 82.84 ± 21.90 μm. The mean thickness of the IPL/INL for each group is as follows: 70.75 ± 8.05, 91.52 ± 21.05, 111.41 ± 27.51, 82.36 ± 11.43 and 98.31 ± 27.94 μm in the control group and groups 1A, 1B, 2A and 2B, respectively. The mean thickness of the OPL/ONL (medium gray bar graph) was 132.37 ± 53.52 μm. The mean thickness of the OPL/ONL for each group is as follows: 105.02 ± 11.25, 175.78 ± 64.02, 199.83 ± 80.24, 135.09 ± 44.43 and 128.42 ± 24.86 μm in the control group and groups 1A, 1B, 2A and 2B, respectively. The mean thickness of the inner/outer layer (dark gray bar graph) was 215.16 ± 69.11 μm. The mean thickness of the inner/outer layer for each group was 175.67 ± 76.57, 267.30 ± 78.09, 311.24 ± 92.99, 217.44 ± 49.74 and 226.73 ± 46.25 μm in the control group and groups 1A, 1B, 2A and 2B, respectively

The mean logMAR BCVA at baseline was 0.16 ± 0.28. Among all groups, the BCVA in the control group (0.07 ± 0.17) was better than that in the other groups, and the BCVA in group 1B (0.36 ± 0.38) was worse than that in the other groups. In the DME groups, no significant difference in BCVA was found between groups 1A, 1B, 2A and 2B (ANOVA, *P* = 0.516) (Fig. 1a). A similar trend was observed only when patients with pachychoroid phenotypes were analyzed (Fig. 1a); the BCVA in the control group (0.08 ± 0.23) was better than that in the other groups, and the BCVA in group 1B (0.37 ± 0.46) was worse than that in the other groups. In DME eyes, no significant difference in BCVA was found between groups 1A, 1B, 2A and 2B (Kruskal-Wallis test, *P* = 0.107).

### Comparison of CMT, SFCT and inner and outer retina layer thicknesses among the groups with pachychoroid features

CMT and SFCT in all groups are shown in Table 2. When only eyes with pachychoroid phenotypes from DME patients were analyzed (groups 1A, 1B, 2A and 2B), CMT was greatest in group 1B (431.00 μm, 500.48 μm, 345.38 μm and 402.95 μm in groups 1A, 1B, 2A and 2B, respectively, *P* < 0.001). A significant difference in CMT was found between all groups except between groups 1A and 2B and between groups 2A and 2B (*P* = 0.831, 0.164, respectively) (Table 2). No significant difference in SFCT was found among groups 1A, 1B, 2A and 2B (*P* = 0.297) (Table 2).

**Table 2:** Comparison of central macular thickness (CMT) and subfoveal choroidal thickness (SFCT)

	Control (***n*** = 285)	Group 1A (***n*** = 37)	Group 1B (***n*** = 43)	Group 2A (***n*** = 70)	Group 2B (***n*** = 60)	***p***-value	Total (***n*** = 495)
**All eyes**							
**CMT (**μm **± SD)**	267.29 ± 24.44	445.89 ± 133.11*	532.47 ± 161.65*†	336.21 ± 82.93*†‡	408.22 ± 106.32*‡§	**< 0.001**	330.50 ± 117.30
**SFCT (**μm **± SD)**	273.41 ± 95.82	320.89 ± 71.97*	310.70 ± 82.04	296.14 ± 87.52	279.25 ± 100.16	**0.006**	284.12 ± 93.54
**Eyes with pachychoroid**							
**CMT (**μm **± SD)**	265.92 ± 24.57	431.00 ± 139.97*	500.48 ± 169.68*†	345.38 ± 80.82*†‡	402.95 ± 105.92*‡§	**< 0.001**	340.90 ± 124.80
**SFCT (**μm **± SD)**	374.33 ± 56.20	360.08 ± 35.11	357.59 ± 56.74	370.56 ± 55.67	388.14 ± 70.68	0.297	371.28 ± 55.82

*Statistically significant (*p* < 0.05) compared to control group

†Statistically significant (*p* < 0.05) compared to group 1A

‡Statistically significant (*p* < 0.05) compared to group 1B

§Statistically significant (*p* < 0.05) between group 2A vs 2B

*SD* standard deviation

One-way ANOVA and post hoc tests (CMT, SFCT) were used

Bold values denote statistical significance (*p* < 0.05)

Among the eyes with pachychoroid phenotypes, IPL/INL and OPL/ONL thicknesses were greatest in group 1B (91.52 μm, 111.41 μm, 82.36 μm and 98.31 μm in groups 1A, 1B, 2A and 2B, respectively, *P* < 0.001 for IPL/INL thickness; 175.78 μm, 199.83 μm, 135.09 μm and 128.42 μm in groups 1A, 1B, 2A and 2B, respectively, Kruskal-Wallis test, *P* < 0.001 for OPL/ONL thickness) (Fig. 1b). In group 1, no eyes with focal DME (26 eyes) or diffuse DME (27 eyes) showed normal neuroretinal thickness (within the 3000-μm central subfield) above the SMD. The highest point of thickening within the 3000-μm central subfield in the neural retina was significantly higher in eyes with SMD (groups 1A and 1B) than that in eyes without SMD (groups 2A and 2B) (Fig. 1b).

Intriguingly, in group 2A, definite microaneurysms resulting in OPL/ONL edema were not identified in 13 eyes among 32 eyes with pachychoroid phenotypes (40.6%) by OCT and FA (Table 3). This percentage was higher than that of eyes with no identified microaneurysms and without pachychoroid phenotypes in group 2A (5 among 38 eyes [13.2%] without pachychoroid phenotypes in group 2A had no definite microaneurysms) (Pearson’s chi-square test, *P* = 0.009). Among 38 eyes without pachychoroid features, 33 eyes with microaneurysms showed higher CMT than did 5 eyes without definite microaneurysms (342.79 and 251.20 μm, *P* = 0.010), confirming that leakage from microaneurysms contributed to macular edema in these patients. Among 18 eyes without definite microaneurysms, 13 eyes with pachychoroid features showed higher CMT than did 5 eyes without pachychoroid features (358.23 and 251.20 μm, *P* = 0.019). When we analyzed 52 eyes with microaneurysms or 52 eyes in which we considered that a leak from microaneurysms was the major mechanism of their macular edema (focal-type edema), the CMT of 19 eyes with pachychoroid features was not different from that of 33 eyes without pachychoroid features (336.58 and 342.79 μm, *P* = 0.676). However, BCVA was better in 19 eyes with pachychoroid features than in 33 eyes without pachychoroid features (0.02 and 0.20, *P* = 0.044). Finally, among 32 eyes with pachychoroid features, no differences were found in CMT or other OCT parameters measured between 13 eyes without definite microaneurysms and 19 eyes with microaneurysms (Table 3).

**Table 3:** Comparison of the presence of microaneurysm (Ma) in group 2A

	Group 2A (***n*** = 70)							
	Non-pachychoroid type (***n*** = 38)		Pachychoroid type (***n*** = 32)					
	Ma (−) (***n*** = 5)	Ma (+) (***n*** = 33)	Ma (−) (***n*** = 13)	Ma (+) (***n*** = 19)	***p***-value*	***p***-value†	***p-***value‡	Total (***n*** = 70)
**CMT (**μm **± SD)**	251.20 ± 67.58	342.79 ± 81.28	358.23 ± 115.08	336.58 ± 47.11	0.061	**0.010**	**0.019**	337.43 ± 166.61
**SFCT (**μm **± SD)**	219.80 ± 69.90	236.24 ± 52.27	384.46 ± 64.86	361.05 ± 47.92	**< 0.001**	0.645	**< 0.001**	296.47 ± 87.46
**Thickness of INL/IPL (**μm **± SD)**	401.40 ± 37.45	424.88 ± 151.43	431.77 ± 73.20	398.11 ± 39.54	0.831	0.967	0.703	417.21 ± 110.65
**Thickness of ONL/OPL (**μm **± SD)**	609.80 ± 86.02	665.91 ± 160.43	715.92 ± 291.83	647.74 ± 161.88	0.862	0.769	0.443	666.26 ± 186.55
**BCVA (logMAR) (mean ± SD)**	0.15 ± 0.31	0.20 ± 0.34	0.23 ± 0.22	0.02 ± 0.26	0.099	0.331	0.387	0.15 ± 0.30

*Ma* microaneurysm, *CMT* central macular thickness, *SFCT* subfoveal choroidal thickness, *INL* inner nuclear layer, *IPL* inner plexiform layer, *ONL* outer nuclear layer, *OPL* outer plexiform layer, *BCVA* best-corrected visual acuity

Symbols depict the *p*-values between the total group (*), the non-pachychoroid type without Ma, the pachychoroid type with Ma(†) and the pachychoroid types with and without Ma (‡)

The Kruskall-Wallis test with the Mann-Whitney U test was applied

Bold values denote statistical significance (*p* < 0.05)

### Findings on ICGA and OCTA in diabetic eyes with pachychoroid features

Among 106 eyes with pachychoroid phenotypes (106 DME eyes with pachychoroid phenotypes in groups 1A, 1B, 2A and 2B) and ICGA results, 47 eyes showed early hypofluorescent lesions in the early phase and choroidal hyperpermeability in the late phase. Good-quality OCTA images of the CC slab were obtained for 49 eyes among the 106 eyes. In groups 1A, 1B, 2A and 2B, 68.75, 40.00, 42.86 and 66.67% of eyes showed areas devoid of flow at the CC slab on OCTA, respectively (Supplemental Fig. 1, 2, 3, 4).

## Discussion

In the present study, pachychoroid phenotypes were more frequent in diabetic eyes with SMD than in those without SMD. We also observed a certain type of focal edema that might be derived from direct RPE leakage and is associated with pachychoroid phenotypes (Supplemental Fig. 1 and 3). Progressive thickening of the choroid or a thickened choroid with the progression of DR or the presence of DME has been reported [8, 13, 20]. SMD is reportedly related to increased CT, increased CC permeability and outer BRB dysfunction. Based on the findings of the present study, we suggest that there may be specific types of DME associated with pachychoroid characteristics in addition to CT changes along with DR and/or DME. This idea can also explain the very divergent reports regarding CT and DR and/or DME thus far [12–20]. Proteinaceous exudate in the subretinal space might be at least partially caused by excessive leakage from the CC.

When we analyzed eyes with pachychoroid phenotypes in the control and groups 1A, 1B, 2A and 2B, no significant difference in SFCT or BCVA was found between group 1B and group 2B. However, CMT and the thickness of the inner/outer retinal layers were significantly higher in group 1B (all *P* < 0.001). Additionally, no difference in SFCT or BCVA was found between group 1A and group 2A. Nevertheless, CMT and the thickness of the inner/outer retinal layers were significantly larger in group 1A than in group 1B (all *P* < 0.001). Thus, SMD may develop with large neural retinal fluid accumulation, but no correlation was found between the presence of SMD and visual acuity, consistent with findings in other studies [8, 11, 26]. As Gaucher et al. [8] described, functional impairment of the RPE and choroidal changes could lead to SMD in diabetic eyes, in addition to insufficient passive resorption of intraretinal fluid for the development of SMD. Nonperfusion of the CC causes ischemic damage or necrosis of the RPE and breakdown of outer BRB, resulting in the development of SMD [12, 13]. Loss of the CC or decreased choroidal blood flow may result in a hypoxic environment around the RPE, followed by upregulation of VEGF production by the RPE [8, 27]. In histologic studies of the diabetic choroid, choroidal neovascularization (CNV) that was associated with areas of CC loss at or peripheral to the equator was observed [28]. FA and ICGA of these patients show filling delay of the choroid, indicating CC nonperfusion in the early phase and multiple hyperfluorescent spots in the middle to late phase [29]. In our study, macular ischemia, which appears as decreased CC flow on OCTA, was detected in 14 (42.86%) and 9 (66.67%) eyes in groups 1A and 1B, respectively. As Gaudric et al. [30] hypothesized, if choroidal ischemia is moderate, passage of fluid through the RPE could lead to SMD; in contrast, RPE cell death occurs with a greater extent of choroidal ischemia.

Interestingly, among 18 eyes without definite microaneurysms in group 2A (the focal edema group), we noted that 13 eyes with pachychoroid features showed greater CMT than that in 5 eyes without pachychoroid features (358.23 and 251.20 μm, *P* = 0.019, Table 3), suggesting that an RPE leak accompanied by pachychoroid phenotypes may be a major contributing mechanism for macular edema in these patients. No difference in BCVA between these two subgroups was found (*P* = 0.387, Table 3). When we analyzed 52 eyes with microaneurysms in which a leak from microaneurysms was the major mechanism of macular edema, there was no difference in CMT between 19 eyes with pachychoroid features and 33 eyes without pachychoroid features (336.58 and 342.79 μm, *P* = 0.676). However, BCVA was better in 19 eyes with pachychoroid features than in 33 eyes without pachychoroid features (0.02 and 0.20, *P* = 0.044). These findings collectively suggest that eyes with edema from an RPE leak in pachychoroid phenotypes could develop during the course of DR. Thus, these features might not necessarily indicate a chronic course or a poorer prognosis. Remarkably, 7 of 18 eyes without definite microaneurysms in group 2A showed intraretinal fluid accumulation in the nasal macula (Supplemental Fig. 3). Regarding the possible mechanism associated with this unusual location of macular edema in eyes with DME, these eyes may share overlapping features with peripapillary pachychoroid syndrome (PPS), a recently described type of pachychoroid spectrum disease [31]. Although 4 of 16 cases with PPS in the study by Phasukkijwatana et al. [31] had underlying DM, it remains unclear whether DM and/or DME with pachychoroid features can be included in the clinical spectrum of PPS, which is characterized by peripapillary choroidal thickening associated with nasal macular intraretinal and/or subretinal fluid and occasional disc edema. Although the pathophysiological mechanisms of PPS are not completely known [29], similar or shared mechanisms of RPE dysfunction and subsequent fluid leakage into the intraretinal space due to high hydrostatic pressure from peripapillary choroidal congestion are likely operating in our nasally located focal DME eyes with pachychoroid features and without microaneurysms.

This study has several limitations. First, the study design is retrospective, and only images and data collected at the initial presentation were analyzed. Future longitudinal studies including the association of changes in OCT parameters after the treatment of DME are needed. However, the strengths of the present study included a large number of consecutive DR patients in one tertiary hospital. In addition, DME patients were divided into four groups, and the analysis was further conducted on subgroups according to the presence or absence of pachychoroid phenotypes for the first time. Second, the definition of pachychoroid phenotypes has not been clarified; however, we have defined them as defined in previous studies as much as possible [21–25]. Finally, OCTA examination was not conducted on all patients; thus, the definite relationship between CC ischemia and outer retinal damage or edema should be evaluated in future studies. In addition, OCTA could possibly be used to find MA that were not observed in OCT or FA. However, a recent study reported that FA counted a significantly higher number of MAs compared with the five different OCTA devices, including ours (Spectralis OCTA, Heidelberg, Germany) [32]. When compared with FA, Spectralis OCTA showed a sensitivity of 43%, specificity of 55%, positive predictive value (PPV) of 81% and negative predictive value (NPV) of 17%. Due to the lower PPV and NPV, OCTA still has weaknesses in defining the presence or absence of MAs. Another study reported that MAs that appear hyporeflective on structural OCT have a lower detection rate on OCTA [33]. Nevertheless, the possibility of finding new MAs with OCTA exists, and large population studies will be needed in the future.

## Conclusions

In conclusion, SMD and focal edema from RPE leakage may be clinical manifestations of DME with pachychoroid phenotypes. A better understanding of choroidal disturbances in DME is important for accurate evaluation and treatment of DME.

## Supplementary information

**Additional file 1: Figure S1.** A representative case of the focal-edema type with serous macular detachment (group 1A).

**Additional file 2: Figure S2.** A representative case of the diffuse-edema type with serous macular detachment (group 1B).

**Additional file 3: Figure S3.** A representative case of the focal-edema type without serous macular detachment (group 2A).

**Additional file 4: Figure S4.** A representative case of the diffuse-edema type without serous macular detachment (group 2B).

## Data Availability

All data generated or analyzed during the current study are available from the corresponding author on reasonable request.

## Abbreviations

DME: Diabetic macular edema
DR: Diabetic retinopathy
CME: Cystoid macular edema
SMD: Submacular detachment
OCT: Optical coherence tomography
FA: Fluorescein angiography
BRB: Blood-retinal barrier
INL: Inner nuclear layer
IPL: Inner plexiform layer
OPL: Outer plexiform layer
CMT: Central macular thickness
ELM: External limiting membrane
RPE: Retinal pigment epithelium
RVO: Retinal vein occlusion
CT: Choroidal thickness
SFCT: Subfoveal choroidal thickness
NRT: Neural retina layer thickness
EDI OCT: Enhanced depth imaging OCT
PPE: Pachychoroid pigment epitheliopathy
PNV: Pachychoroid neovasculopathy
CSC: Central serous chorioretinopathy
CC: Choriocapillaris
ILM: Internal limiting membrane
PPS: Peripapillary pachychoroid syndrome
PPV: Positive predictive value
NPV: Negative predictive value
