# Supplementary figures and images for: Diabetic macular edema with pachychoroid features
Source: BMC Ophthalmol. 2020 Oct 2;20:392. doi: 10.1186/s12886-020-01663-y (PMC7532553; doi:10.1186/s12886-020-01663-y)

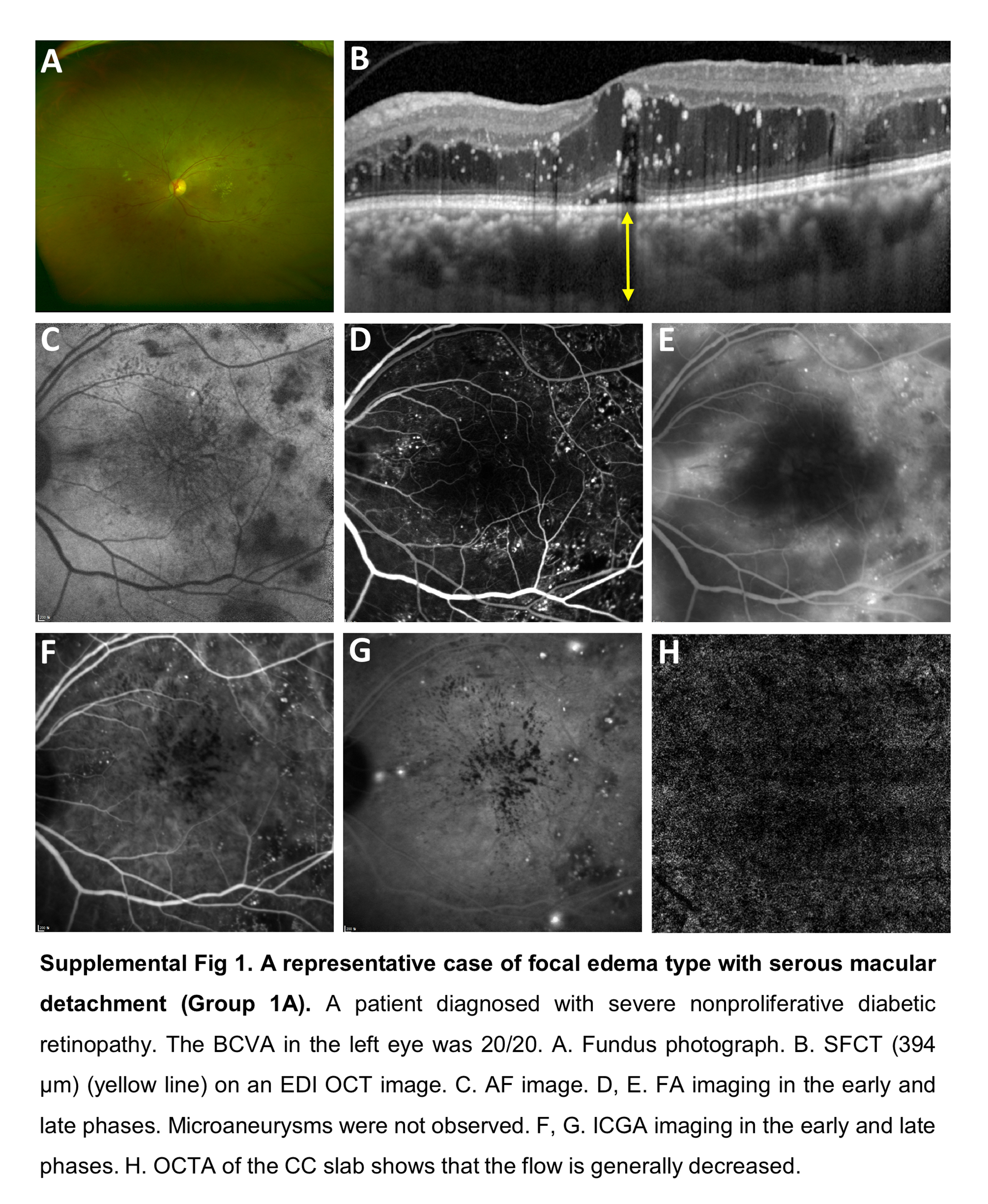

Supplement: Supplementary file 1 — Additional file 1: Figure S1. A representative case of the focal-edema type with serous macular detachment (group 1A). [file 12886_2020_1663_MOESM1_ESM.png]

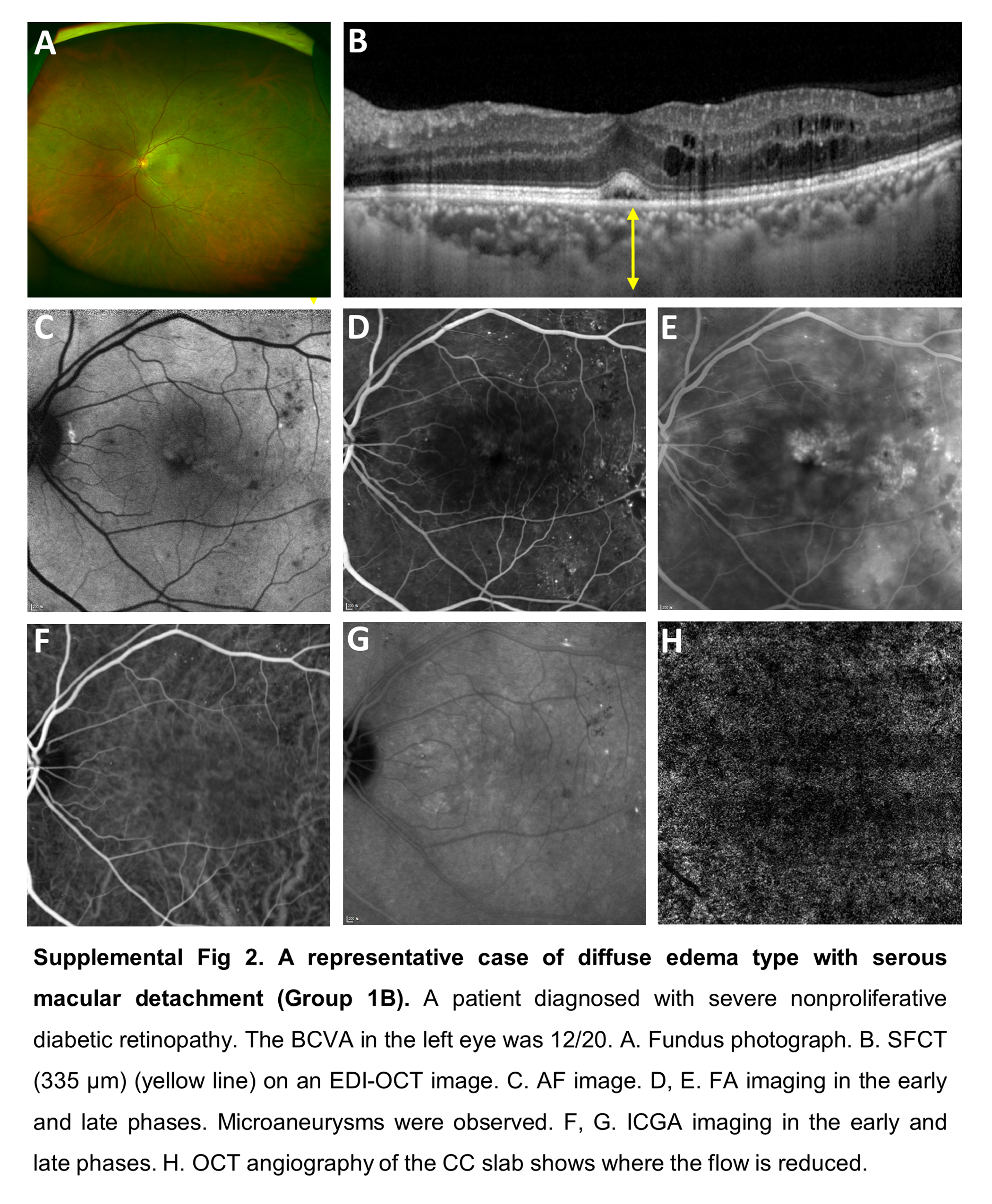

Supplement: Supplementary file 2 — Additional file 2: Figure S2. A representative case of the diffuse-edema type with serous macular detachment (group 1B). [file 12886_2020_1663_MOESM2_ESM.png]

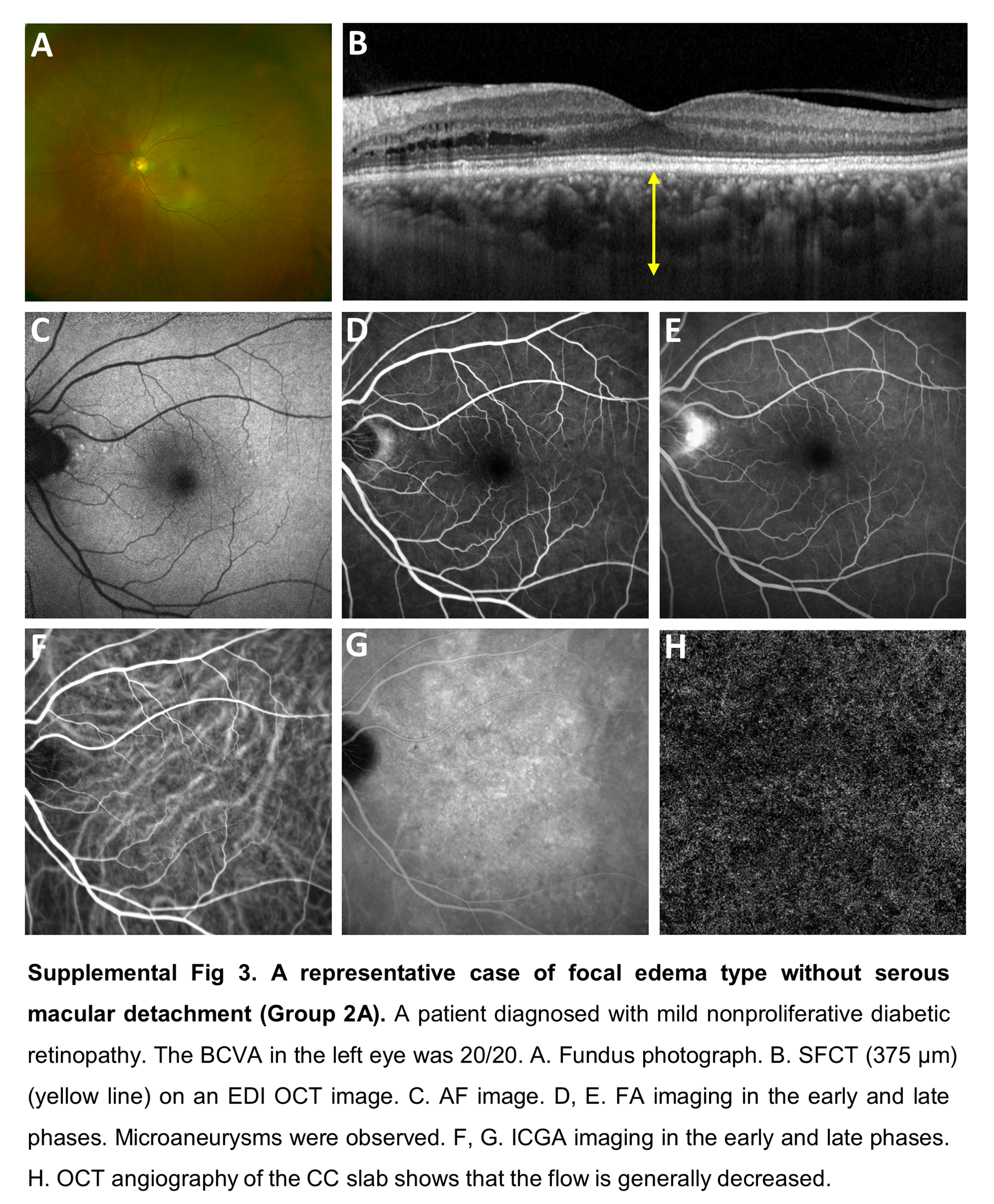

Supplement: Supplementary file 3 — Additional file 3: Figure S3. A representative case of the focal-edema type without serous macular detachment (group 2A). [file 12886_2020_1663_MOESM3_ESM.png]

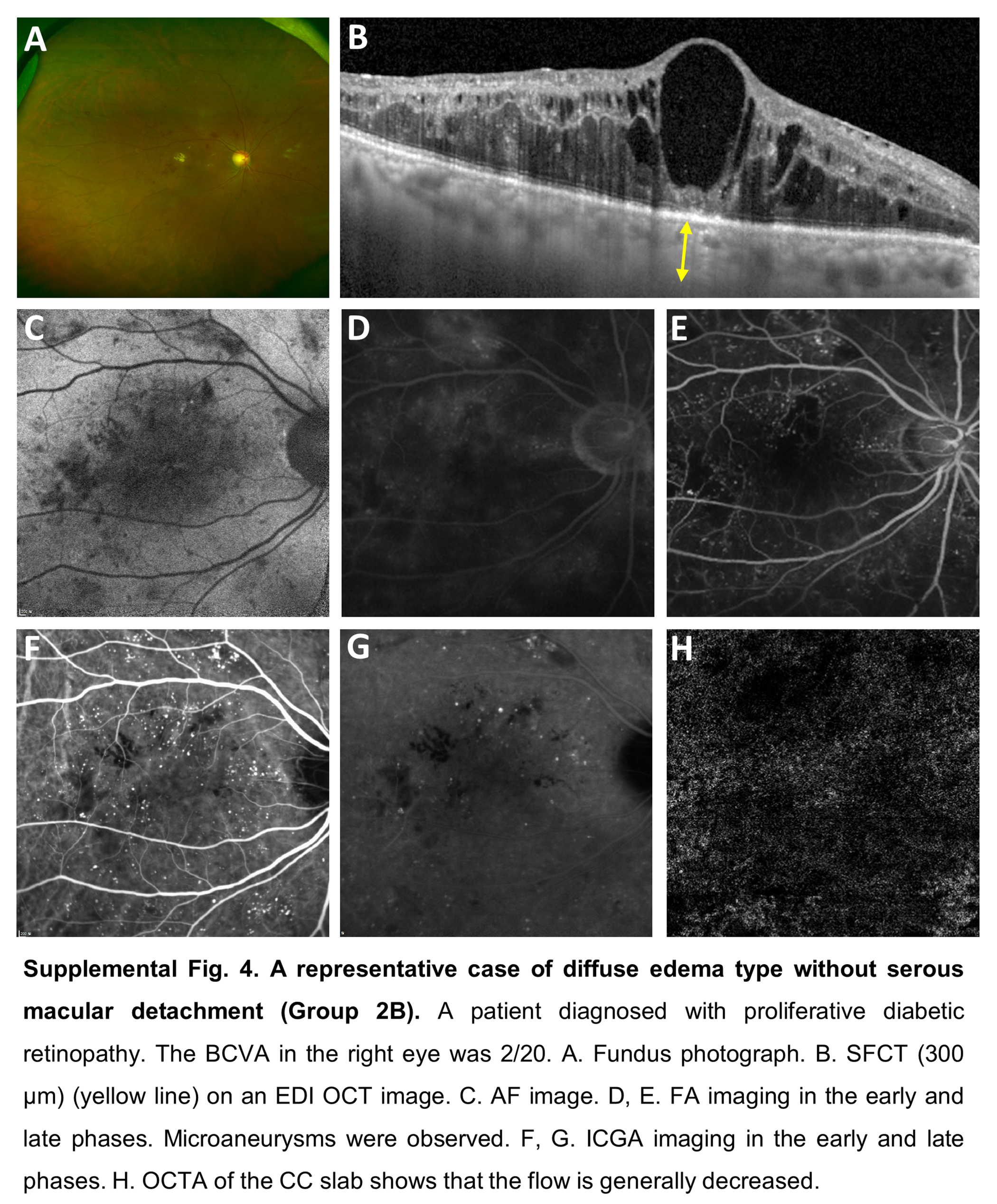

Supplement: Supplementary file 4 — Additional file 4: Figure S4. A representative case of the diffuse-edema type without serous macular detachment (group 2B). [file 12886_2020_1663_MOESM4_ESM.png]
